# Is fiscal deficit ‘curse’ or ‘haven’ for environmental quality in India? Empirical investigation employing battery of distinct ARDL approaches

**DOI:** 10.1016/j.heliyon.2023.e20711

**Published:** 2023-10-10

**Authors:** Mohammad Asif, Vishal Sharma, Hari Prapan Sharma, Hamad Aldawsari, Showkat Khalil Wani, Sunil Khosla, Vinay Joshi Chandniwala

**Affiliations:** a College of Administrative and Financial Science Saudi Electronic University, Riyadh, 11673, Saudi Arabia; b School of Commerce and Economics, Presidency University, Bengaluru, Karnataka, India; c Institute of Business Management, GLA University, Mathura, India; d School of Commerce and Economics, Presidency University, Bengaluru, Karnataka, India; e School of Social Sciences and Humanities, VIT-AP University, Amaravati, India

**Keywords:** Environmental deterioration, Fiscal policy, Cointegration, MTNARDL, India

## Abstract

Undoubtedly, throughout the past half-century, environmental quality has emerged as a significant obstacle to both economic and social endeavors. Recent local and international policy debates have focused on environmental deterioration and global warming, but how governments balance economic growth and environmental sustainability is still enigmatic. For this reason, we have examined the determinants of environmental quality in India from 1972 to 2021. More specifically, we have investigated whether the fiscal deficit is ‘curse’ or ‘haven’ for environmental quality (CO2) in India. Moreover, this study deliberated four other predictors, comprising technological development (TIN), fossil fuel consumption (FFC), urbanization (Ub), and human capital index (HCI). In order to attain this objective, a range of econometric estimation techniques are employed to ensure the validity and reliability of the outcomes. For instance, we have employed a battery of ARDL approaches, such as standard ARDL, nonlinear ARDL, and multiple threshold NARDL approaches. In light of our research findings, we will be focusing directly on the examination of the NARDL and MTNARDL outcomes. This is due to the empirical evidence indicating the existence of asymmetric effects resulting from FD on CO2 emissions in India. The NARDL approach reveals that the consequence of fiscal deterioration is more pronounced, and the influence of fiscal progress is mild in terms of CO2 emission growth. Further, the outcomes of the MTNARDL approach revealed that the size of the extremely low changes in FD is much higher than the extremely high changes in FD in both models. This implies that as the FD rises, CO2 ascends more significantly, and when the FD lowers, CO2 declines progressively. In a nutshell, FD has a long-run positive and asymmetric impact on CO2 in India; thus, we may conclude that FD is considered the ‘curse’ for CO2 in India. Furthermore, TIN, HCI, and Ub have detrimental effects on CO2, whereas FFC stimulates CO2 in India. This research work provides some important policy implications for environmentalists, economists and macroeconomic policymakers to promote a green and healthy environment.

## 1 Introduction

The stakes of environmental issues have never been higher in human history as they are now [[1], [2], [3], [4]]. The main reasons are climate change, the loss of biodiversity, the acidification of the oceans, deforestation, the pollution of the oceans with plastic, and a lack of available water [[4], [5], [6]]. The process of economic development promises to elevate the standard of living, yet concurrently engenders environmental deterioration [[7], [8], [9], [10]]. Protecting the environment and sustainable economic growth are the two main issues that developing countries have to confront [11]. In addition to having a direct impact on human health, environmental degradation also has an impact on glacier melting, wildlife, rainfall, and agriculture [12]. According to the Global Energy and CO2 Status Estimate Report China, the United States, the EU27, and India are said to be responsible for 85 % of the net rise in global emissions [13,14]. In 2021, Asian countries accounted for 53 % of global emissions and 60 % of the world's population [15]. The Chinese economy contributes 31 % (10.66 billion metric tonnes CO2) to global emissions, followed by the US at 13 % (5.3 billion metric tonnes CO2) and the EU27 at 7.5 % (2.9 billion metric tonnes CO2). India is the fourth-largest worldwide emitter, with 7.02 % (2.6 billion metric tonnes CO2) of global emissions in 2021. Industrial activity during the last decades has created greenhouse gas (GHG) buildup in the atmosphere, which contributes to climate change [4]. Nations have sought economic growth since the industrial revolution. The competition has resulted in a previously unprecedented surge in greenhouse gas emissions, particularly carbon, causing global warming and ozone layer depletion. Several scientific studies have backed nation-wide CO2 emission reduction plans [16]. Environmental risks are a source of concern for everyone, as well as a factor in political, social, and economic decisions [17,18]. Recent local and international policy debates have focused on environmental deterioration and global warming, but the important question is how the governments balance economic growth and environmental sustainability, which is still enigmatic [11].

Fiscal policy tools, namely taxes and government expenditures, exhibit a direct correlation with GDP expansion, production capacity, energy consumption, and environmental well-being [6]. Fiscal policies have the potential to facilitate the establishment of environmentally sustainable and resilient development with low carbon emissions [19,20]. The post-Keynesian school of thought places significant emphasis on the utilisation of fiscal policy as a means of achieving both macroeconomic stability and high levels of employment [[21], [22], [23]]. (Dafermos et al., 2018; Tcherneva, 2014; Fazzari, 1994). The implementation of fiscal policy by governmental entities for the purpose of revenue collection and expenditure is closely linked to environmental sustainability in countries with high carbon emissions, including but not limited to China, the United States, and India [11]. noted that fiscal policy shocks have significant short-term increases in carbon emissions while having significant long-term decreasing effects on environmental pollution. In the event of an expansionary policy, overall demand within the economy will rise, leading to an increase in derived demand. The escalation of derived demand is anticipated to result in elevated carbon emissions and degradation of the environment [24]. The implementation of a contractionary policy is expected to result in a reduction in the derived demand and emission levels [[24], [25], [26]]. As a result, research into how fiscal policy affects the environment from a post-Keynesian perspective is still in its very early stages. Therefore, a very interesting field of research is examining the connection between fiscal policy tools and environmental degradation.

The allocation of government expenditure should be optimized by directing resources towards specific divisions where spending is deemed necessary [6]. Moreover, analysis indicates that investing in public goods can result in a more significant decrease in CO2 emissions compared to investing in aggregate emissions from both consumption and production [27]. Fiscal policies focused on escalating and implementing adaptive measures are necessary for ecological sustainability [28]. So, taxation as a tool of fiscal policy can increase the efficiency of energy, and tax incentives have a positive and important effect on the quality of the environment [19,29,30]. Fiscal policy has an impact on income growth, which has consequences for energy use and creates environmental issues. The increase in energy demand has occurred concurrently with economic expansion, resulting in negative environmental effects. This is primarily attributable to the growing consumption of non-renewable energy sources, which has resulted in an increase in both GDP growth and CO2 emissions [31,32]. [33] claim that fiscal deficit reduction will promote capital accumulation and economic growth. Thus, fiscal policy may indirectly increase energy consumption due to higher economic activity. Currently, the most significant challenges that emerging countries must overcome are those associated with environmental quality and economic expansion. In the context of economic development, environmental degradation in developing nations is one of the most prominent issues.

The link between the fiscal deficit and the amount of carbon emissions (CO_2_) has become an important issue of research and a subject of discussion [11,34]. Given the focus on creating a sustainable environment and the critical role that governments play in achieving this goal, it is worth noting that when a government runs a fiscal deficit, it may need to borrow money to finance its spending [35]. A high fiscal deficit can lead to cuts in public spending, including spending on environmental protection measures. This can result in reduced investment in clean energy, lower funding for environmental research, and fewer resources allocated for pollution control and monitoring. The fiscal policies that prioritize short-term economic growth over environmental protection may result in higher CO2 emissions in the long run. For example, subsidies for fossil fuels can incentivize their use and discourage investment in renewable energy. In this context, few studies have examined the impact of fiscal policy instruments on carbon emissions, highlighting the crucial role of fiscal policy in promoting environmental sustainability [30,36].

In addition to fiscal policy, a wide range of other macroeconomic variables have been identified as potential causal factors for carbon dioxide (CO2) emissions, operating through various transmission mechanisms [37]. Several empirical studies have examined the potential relationship between government expenditure and environmental quality [[38], [39], [40]]. According to the study conducted by Ref. [41], four primary transmission mechanisms were identified to explain how the level and structure of fiscal spending can impact pollution levels. These mechanisms include scale, composition, technique, and income effects. According to Ref. [42], the primary factors contributing to the degradation of environmental quality are the enhanced economic system, industrialization, and Ub observed in both developing and developed nations [42]. also suggested that Ub in countries with the highest healthcare expenditures that makes efficient use of energy conservation and eco-friendly technologies contributes significantly to the improvement of environmental quality. As the level of Ub rises, the rural population migrates to urban areas in pursuit of a higher level of education. As the quality of human resources improves, higher human standards will displace other material resources. However, environmental issues are exacerbated by human activities. The existing literature, such as [[43], [44], [45], [46]], demonstrated a clear association between HCI and energy consumption, indicating the positive effects of HCI on environmental quality. These studies revealed that higher HC stimulates environmental consciousness and adherence, resulting in less environmental degradation.

Various authors have undertaken research on the relationship between fiscal policy and environmental quality in a linear and non-linear framework [6,47,48]. But in the literature of fiscal policy and environmental quality, the research gap is found in context-limited studies from a specific Indian perspective, and no previous study used a broader multiple threshold NARDL (MTNARDL), which overcomes the limitations of previous methods such as ARDL and NARDL by analyzing the effect of extreme small and large values of variables of concern. Therefore, there is a need to have a clear understanding of the relationship between fiscal deficit and environmental quality in Indian context. The present study fills this gap by investigating the dynamic nexus between fiscal deficit and environmental quality using battery of ARDL approaches in an Indian context. The purpose of this study is to investigate the connection between fiscal deficits and environmental quality while considering the other key variables into consideration, in a regression analytical model to get reliable estimates in a multivariate analytical framework. The specific objectives are as follows:

• To investigate the short run and long run associations between fiscal deficit and environmental quality, in the presence of other control variables.
• To measure the impact of fiscal deficit on environmental quality in a linear and nonlinear framework in the Indian context.

This study extends the literature on fiscal deficit and environmental quality in multiple ways. The originality and innovation of the present study lie in the application of very advanced version of ARDL model i.e., multiple threshold NARDL (MTNARDL) model. According to Ref. [49], the multiple threshold framework is more effective at capturing nonlinear transmission than the single threshold model. Second, in contrast to the existing literature, which have taken the predictors of environmental quality in targeted manners, this study innovatively integrates all the important variables, such as technological development, fossil fuel consumption, human capital index, and urbanization, within the same analytical framework. Thirdly, in is the case of the Indian economy, the relationship between fiscal policy and environmental quality very limited studied by research. Being the fourth largest economy, understanding the determinants, status, and capacity to reduce environmental degradation in India is crucial from the perspective of the global economy. Understanding the impact of fiscal policy on environmental quality for emerging economies such as India, the most populous country in the world, will aid in achieving the SDGs and generalising the outcome for other economies with comparable characteristics.

## 2 Literature review

Numerous studies have shown that a number of macroeconomic factors, including energy use, urbanisation, trade openness, and financial development, have a significant impact on environmental deterioration [35,[50], [51], [52], [53], [54]]. This section has presented the previous related literature on the nexus between the fiscal deficit and CO_2_ emissions, and the nexus between other covariates and CO_2_ emissions.

### 2.1 Fiscal deficit and carbon emission (CO_2_) nexus

Numerous studies are available that have examined the nexus between the fiscal deficit and CO_2_ across the globe [30]. claimed that fiscal policies have significant potential to reduce carbon emissions. There is a bidirectional causal relationship between global per capita carbon emissions and economic growth [55]. [36] illustrate that fiscal aggregates can contribute to the decrease in carbon emissions, underscoring the vital role of fiscal policy in promoting environmental sustainability. While [56] have uncovered that over time fiscal policy instruments significantly worsen the quality of the environment and concluded that an expansionary fiscal policy poses a threat to environmental quality in China. A recent study by Ref. [57] found that expansionary fiscal policy exacerbates the negative effects of CO2 emissions. However, contractionary fiscal policy is an effective means of mitigating CO2 emissions negative effects [34]. demonstrate a correlation between government expenditures and environmental deterioration [58]. argued that taxation plays a vital function not only in the economy but also in concerns pertaining to the environment. This is due to the fact that taxes are inversely linked to energy consumption, which in turn affects carbon emissions. Although the results of various studies revealed that the effects of a carbon tax have been inconsistent, taxation is still regarded as the most effective instrument for environmental policies, as reported by Ref. [59]. According to Ref. [60], the adoption of fiscal and tax incentives is beneficial for countries to enhance their energy efficiency. But [59] suggest that for lower greenhouse gas levels, only a few countries have changed their policies related to the environment towards carbon taxes [11]. indicate that a positive surge in government expenditure has an adverse effect on environmental quality in developing countries such as China, India, Malaysia, Indonesia, Iran, Turkey, Thailand, and the UAE, while for Japan, a positive effect of government expenditure on environmental quality has been reported. Conversely, the results indicate that reducing government expenditure would have a positive effect on environmental quality in these economies, with the exception of Japan, where it would have a negative impact.

### 2.2 Covariate influencing carbon emission (CO_2_)

The literature examining the covariates that influence CO2 is categorized into four strands: the Ub-CO2 nexus, FFC-CO2 nexus, TIN-CO2 nexus, and HCI–CO2 nexus. The first strand of literature outlines the empirical link between Ub and CO_2_. The empirical findings have revealed a two-fold relationship: a positive and negative correlation between Ub and CO_2_ [[61], [62], [63], [64], [65], [66]]. According to the findings of [66], Ub strengthens the positive association between economic growth and both carbon emissions and ecological footprint. According to the findings of [67], Ub has been found to mitigate carbon emissions originating from the transportation sector. However, it is important to note that the impact of Ub on carbon emissions is comparatively less significant when compared to the influence of energy efficiency [68]. found that factors such as the rate of Ub, population agglomeration, economic development, industrial development, urban construction, and transportation construction have contributed to the exacerbation of environmental pollution. Further, they show that the effects of Ub on environmental pollution exhibit periodic fluctuations. Ub has led to an increased demand for energy consumption in both industrialised and populous nations, and this phenomenon is strongly correlated with higher income levels, which lead to increased consumption, which has a consequence for environment.

The nexus between TIN and the environment has been studied from two perspectives, one being the potential for technological innovations to enhance environmental sustainability. For instance, in Malaysia [69], concluded that TIN reduces carbon emissions due to the country's green and eco-friendly technology [70]. for selected European nations and [71] for G-7 countries also found similar findings, where TIN was very important in lowering environmental damage based on consumption [72]. indicate that green patent applications have been shown to play a crucial role in reducing carbon emissions from the environment [73]. studied 280 cities in China and found that TIN initially has an incremental impact on the environmental footprint, but after reaching a threshold point, it suppresses the environmental footprint [74]. applied the STIRPAT model to examine West Asia and Middle East nations and found that TIN can enhance environmental sustainability by reducing the environmental footprint. Meanwhile [75], show that TIN efficiently shrinks CO2 emissions but is ineffective in shrinking the environmental footprint for Big Emerging Markets [76]. conducted a study on 73 developing countries and found that TIN helps mitigate the environmental footprint and improves the adverse environmental impacts linked with natural resource usage [77]. established that TIN was harmful to the ecological system by boosting the environmental footprint of Asia Pacific Economic Cooperation (APEC) countries. In summary, there is no consensus regarding the impact of TIN on environmental quality.

The third strand of literature discusses studies that emphasize the implications of fossil fuel consumption (FFC) on the emission of CO_2_. Most of the studies highlight the positive association between FFC and CO_2_. For example [78], along with other variables, analyzed the relationship between consumption of fossil fuels and carbon emissions in Tunisia. The study found that there is a positive and significant relationship between the two variables [79]. also discovered the positive effect of energy intensity on carbon emissions for various Asian nations [80]. found that per capita energy consumption reduces carbon emissions in nine industrialised nations [81]. showed that hydroelectricity and combustible renewable and waste energy reduce carbon emissions, but fossil fuel power generation increase carbon emissions in Ghana [82]. show that global renewable energy can promote economic growth while improving the environment [83]. found that renewable energy decreases pollution in 14 sub-Saharan African nations, but nonrenewable energy increases it [84]. found that renewable energy consumption and human capital have heterogeneous effects on carbon emissions at before- and after-EKC turning points. Human capital has a better emission reduction effect for countries after the EKC turning point. According to Ref. [85], the significant consumption of fossil fuels in China can be primarily attributed to the country's remarkable economic growth.

The last strand discussed the relationship between human capital (HC) and CO_2_. The study that emphasized the nexus between human capital and CO_2_ discloses a mixed finding between HC and CO_2_ emissions [86]. employed the quantile ARDL approach and found a negative association between HC and CO_2_ emissions in China [86]. applied both NARDL and 2SLS econometric methods, and their findings indicate that G-7 countries also exhibit similar outcomes. While for India [87], observed a negative correlation between HC and EF using ARDL and VECM models [88]. applied the ARDL approach and found that HC leads to a reduction of EF in Pakistan. In the case of the US [89], observed a similar findings: HC is negatively associated with EF [90]. report similar results in their study of China, while [91] observe comparable findings in G-7 countries. In contrast [62], found a positive correlation between HC and EF in Pakistan. On the other hand [92], did not identify a significant impact of HC on EF in BRICS nations. Based on the literature discussed, we have formulated our hypotheses.

According to the literature, carbon emission is significant implications on environment quality. It has been found that FD, Ub, TIN, FFC and HCI have significant implications on carbon emission in many developing and developed economies. Given the significance of research on understanding the macroeconomic determinants of carbon emissions for a sustainable environment in India. The study addresses the main question, whether fiscal policy has a significant impact on carbon emissions in India. The research model is controlled by including the other variables, namely urbanisation, fossil fuel consumption, technological innovation, and human capital. The following hypothesis has been developed based on the above research questions.

H1 *FD does not have a significant impact on CO*_*2*_*emission in India*

H2 *Urb does not have a significant impact on environmental quality in India*

H3 *TIN does not have a significant impact on environmental quality in India*

H4 *FFC does not have a significant impact on environmental quality in India*

H5 *HCI does not have a significant impact on environmental quality in India*

## 3 Materials and methods

### 3.1 Data set, variables and data sources

The present study investigates whether fiscal deficit is ‘curse’ or ‘haven’ for environmental quality in India. This study utilizes time series data collected annually from 1972 to 2021. More specifically, this study uses carbon dioxide emissions (CO_2_) as the proxy variable for environmental quality. In addition, the study employs five predictor variables, including the FD, Ub, TIN, FFC and HCI. To minimize the dispersion of the data, we have transformed all variables, excluding HCI, into natural logarithms [[93], [94], [95]]. This transformation also facilitates addressing heteroscedasticity and multicollinearity issues [71]. Each variable is listed in Table 1, accompanied by its description, predicted sign, and sources.

**Table 1:** Description of the variables.

Variables	Acron.	Description	Source	Sign
Dependent Variables				
Carbon dioxide	lnCO_2_	Log of Carbon dioxide	BP Statistical Review of World Energy	
*Independent Variables*				
Fiscal Deficit	lnFD	Log of fiscal deficit (Central and State Government)	Reserve Bank of India	+
Technological Development	lnTIN	Log of Technological Development	OECD statistics https://stats.oecd.org/#	–
Fossil Fuel Consumption	lnFFC	Log of Fossil Fuel Consumption	BP Statistical Review of World Energy	+
*Control Variables*				
Human Capital Index	HCI	Human Capital	FRED economic data https://fred.stlouisfed.org	–
Urbanization	Ub	Log of Urbanization	World Development Indicators https://databank.worldbank.org/home.aspx	–

Thus, the current study has categorized the functional form and provided the following equation:

$${lnCO}_{2}=f\left(lnFD,lnTIN,lnFFC,HCI,Ub\right)$$

Each variable is described in Table 1.

### 3.2 Methodology and model specification

There are numerous standard methodologies, such as [[96], [97], [98]], for exploring the cointegration or long-run nexus amidst the dependent variable and its covariates [99]. presented the auto-regressive distributed lag (ARDL) strategy to cointegration, which has overcome the relevant empirical constraints intrinsic in conventional approaches.^1^ In order to investigate the linear/symmetric response of CO2 to FD in India, we have therefore implemented the ARDL methodology.

Utilizing the unconstrained error correction model (ECM), this study analyzed the cointegration amongst CO2 and its determining variables in Eq. (1):

(1) $$\Delta {lnCO2}_{t}=\alpha_{0}+\sum_{i=1}^{n}\delta_{i}\Delta {lnCO2}_{t-i}+\sum_{i=0}^{m}\left[\beta_{1}\Delta {lnFD}_{t-i}+\beta_{2}\Delta {lnTIN}_{t-i}+\beta_{3}\Delta {lnFFC}_{t-i}+\beta_{4}\Delta {HCI}_{t-i}+\beta_{5}\Delta {Ub}_{t-i}\right]+\gamma_{1}{lnCO2}_{t-1}+\gamma_{2}{FD}_{t-1}+\gamma_{3}{lnTIN}_{t-1}+\gamma_{4}{lnFFC}_{t-1}+\gamma_{5}{HCI}_{t-1}+\gamma_{6}{Ub}_{t-1}+\mu_{t}$$

where each variable is described in Table 1, Δ describes change or 1st difference; *t* reflects time period; δ_i_ and β_1_ to β_5_ symbolize short-run elasticities; γ_1_ to γ_6_ exhibit long-run elasticities; and μ_t_ is the error term.

F-statistics was considered to assess the null hypothesis stating that there is no cointegration amongst the variables (i.e., H0: γ_1_ = γ_2_ = γ_3_ = γ_4_ = γ_5_ = γ_6_ = 0), as in contradiction of the alternative hypothesis stating that there is cointegration amongst the variables (i.e., H1: γ_1_ ≠ γ_2_ ≠ γ_3_ ≠ γ_4_ ≠ γ_5_ ≠ γ_6_ ≠ 0).

By including the ECM into the analysis, the short-run elasticity parameters are defined in Eq. (2):

(2) $$\Delta \ln\;{CO2}_{t}=\alpha_{0}+\sum_{i=1}^{n}\theta_{i}\Delta {lnCO2}_{t-i}+\sum_{i=0}^{m}\left[\varphi_{1}\Delta {lnFD}_{t-i}+\varphi_{2}\Delta {lnTIN}_{t-i}+\varphi_{3}\Delta {lnFFC}_{t-i}+\varphi_{4}\Delta {HCI}_{t-i}+\varphi_{5}\Delta {Ub}_{t-i}\right]+\psi {ECT}_{t-1}+\mu_{t}$$

Where $\sum_{i=1}^{n}\theta_{i}$ and $\sum_{i=0}^{m}\varphi_{i}$ denotes the short-run coefficients; ECT demonstrates error correction term; and the ECT (ψ) coefficient enumerates the average annual rate of correction. The ARDL method is predicated on the indispensable notion of a symmetric relationship within variables, which necessitates both short- and long-term linear change [100]. affirmed that the NARDL cointegration technique produces advanced outcomes when the link between the endogenous variable and its covariates is asymmetric and regressors have nonlinear ramifications on the effect variable. NARDL is an asymmetric augmentation of the standard ARDL model. The NARDL has significant benefits over mainstream econometric techniques, such as its effectiveness with limited sample sizes and regressors that are not integrated in the same sequence [100],.^2^ This study employs the NARDL methodology in order to analyse the asymmetric consequences of fiscal deficit on India's carbon emissions.

The cointegration test is taken from the subsequent NARDL framework version:

(3) $${lnCO2}_{t}=\alpha_{0}+\beta_{1}^{+}{lnFD}_{t}^{+}+\beta_{2}^{-}{lnFD}_{t}^{-}+\beta_{3}{lnTIN}_{t}+\beta_{4}{lnFFC}_{t}+\beta_{5}{HCI}_{t}+\beta_{6}{Ub}_{t}+\mu_{t}$$

in Eq. (3), the FD is bifurcated into two distinct partial sums (i.e., positive and negative) in Eqs. (4), (5):

(4) $${lnFD}_{t}^{+}=\sum_{j=1}^{t}\Delta {lnFD}_{t}^{+}=\sum_{j=1}^{t}\max\left(\Delta {lnFD}_{j},0\right)$$

(5) $${lnFD}_{t}^{-}=\sum_{j=1}^{t}\Delta {lnFD}_{t}^{-}=\sum_{j=1}^{t}\min\left(\Delta {lnFD}_{j},0\right)$$

Following [[99], [100], [101]] have altered Eq. (3) and reframe it in ARDL form:

(6) $$\Delta {lnCO2}_{t}=\alpha +\rho {lnCO2}_{t-1}+\omega_{1}^{+}{lnFD}_{t-1}^{+}+\omega_{2}^{-}{lnFD}_{t-1}^{-}+\omega_{3}{lnTIN}_{t-1}+\omega_{4}{lnFFC}_{t-1}+\omega_{5}{HCI}_{t-1}+\omega_{6}{Ub}_{t-1}+\sum_{j=1}^{p}\delta_{j}{\Delta lnCO2}_{t-j}+\sum_{j=0}^{q}\left[\theta_{j}^{+}{lnFD}_{t-j}^{+}+\theta_{j}^{-}{lnFD}_{t-j}^{-}+\varphi_{j}{lnTIN}_{t-j}+\varphi_{j}{lnFFC}_{t-j}+\sigma_{j}{HCI}_{t-j}+\gamma_{j}{Ub}_{t-j}\right]+\mu_{t}$$

in Eq. (6), *p* and *q* represent the lag orders of the variables. The coefficients ($\omega_{1}^{+},\omega_{2}^{-},\omega_{3},\omega_{4},\omega_{5},\omega_{6})$ reflects the long-run nexus, whereas, coefficients $\sum_{j=0}^{q-1}{[\theta }_{j}^{+},\theta_{j}^{-},\varphi_{j},{\varphi_{j},\sigma }_{j},\gamma_{j}]$ represents the short-term nexus amongst the variables. However, $\beta_{1}^{+}=\left[-\frac{\omega_{1}^{+}}{\rho }\right],\beta_{2}^{-}=\left[-\frac{\omega_{2}^{-}}{\rho }\right]$ are the long-run asymmetric elasticities for *lnFD*^*+*^ and *lnFD*^*-*^, respectively. $\varepsilon_{t}$ is the white noise.

F-statistics are used to verify the contemporaneous null hypothesis of no cointegration, $\rho =\omega_{1}^{+}=\omega_{2}^{-}=\omega_{3}=\omega_{4}=\omega_{5}=\omega_{6}=0$, when evaluating asymmetric cointegration among variables. The subsequent procedure resembles the ARDL model.

The WALD statistics have been utilized to assess the short- and long-term asymmetries. Lastly, Eqs. (7), (8) represent dynamic multipliers which are derived from Eq. (6):

(7) $$m_{k}^{+}=\sum_{j=0}^{k}\left[\frac{{\partial lnCO2}_{t+j}}{{\partial lnFD}_{j}^{+}}\right]\text{,}$$

(8) $$m_{k}^{-}=\sum_{j=0}^{k}\left[\frac{{\partial lnCO2}_{t+j}}{{\partial lnFD}_{j}^{-}}\right]$$

where, *k* = 0,1,2,3 and $m_{k}^{+}$ and $m_{k}^{-}$ tend towards the respective asymmetric long‐run coefficients $\beta_{1}^{+}=\left[-\frac{\omega_{1}^{+}}{\rho }\right],\beta_{2}^{-}=\left[-\frac{\omega_{2}^{-}}{\rho }\right]\text{,}$ as *k* tends to infinity.

In accordance with [100], [[102], [103], [104]], a single threshold NARDL model was implemented to quantify price transfer asymmetrically. The regressor is segmented into its positive and negative partial sum series within a single threshold NARDL. The positive and negative partial sum series represent the expansion and contraction of the regressor [105]. modified the single-threshold NARDL model of [100] and applied a two-threshold NARDL model for analyzing the effect of extreme small and large exchange rate variations in Economic and Monetary Union (EMU) exports to the US [105]. established two thresholds for variations in exchange rates at the 30th and 70th quantiles. Additionally [49], developed a broader multiple threshold NARDL (MTNARDL) model. Studying the US dataset from 1996 to 2014 [49], determined that the multiple threshold framework can capture the nonlinear transmission of prices more effectively than the single threshold NARDL model. Therefore, we have also employed the MTNARDL model to examine the response of CO2 due to the extreme changes in FD in India.

Following [105], first, we divide the lnFD into two thresholds as mentioned in Eq. (9):

(9) $${lnFD}_{t}={lnFD}_{0}+\ln\;{FD}_{t}\left(w_{1}\right)+{lnFD}_{t}\left(w_{2}\right)$$

where, ${lnFD}_{t}\left(w_{1}\right)$ and ${lnFD}_{t}\left(w_{2}\right)$ denote the two partial sum series that are set at the 25th and 75th quintiles, which are denoted by $\tau_{25}$ and $\tau_{75}$, respectively, and are calculated as described in Eqs. (10), (11):

(10) $${lnFD}_{t}\left(w_{1}\right)=\sum_{j=1}^{t}{\Delta lnFD}_{j}\left(w_{1}\right)=\sum_{j=1}^{t}{lnFD}_{j}I\left\{{lnFD}_{j}\leq \tau_{25}\right\}$$

(11) $${lnFD}_{t}\left(w_{2}\right)=\sum_{j=1}^{t}{\Delta lnFD}_{j}\left(w_{2}\right)=\sum_{j=1}^{t}{lnFD}_{j}I\left\{{lnFD}_{j}>\tau_{75}\right\}$$

in Eqs. (10), (11), I{T} is an indicator function, which has a value of one, if the conditions given in {} are satisfied; otherwise, it has a value of zero. The MTNARDL model with quintile series of FD can be shown as in Eq. (12):

(12) $$\Delta {lnCO2}_{t}=\alpha_{0}+\sum_{i=1}^{n}\eta_{1,i}\Delta {lnCO2}_{t-i}+\sum_{i=0}^{m}\left(\eta_{2,i}{\Delta lnTIN}_{t-i}+\eta_{3,i}{\Delta LNFFC}_{t-i}+\eta_{4,i}{\Delta HCI}_{t-i}+\eta_{5,i}{\Delta Ub}_{t-i}\right)+\sum_{l=1}^{2}\sum_{j=0}^{p}\eta_{k}{\Delta lnFD}_{t-j}\left(w_{l}\right)+\psi_{1}{lnCO2}_{t-1}+\psi_{2}{lnTIN}_{t-1}+\psi_{3}{lnFFC}_{t-1}+\psi_{4}{HCI}_{t-1}+\psi_{5}{Ub}_{t-1}+\sum_{l=1}^{2}\psi_{k}{lnFD}_{t-1}\left(w_{l}\right){+\varepsilon }_{t}$$

where k = 1 + 3.

Here, to assess the comprehensive co-integration, we formulated the null hypothesis of no co-integration (H0: $\psi_{1}=\psi_{2}=\psi_{3}=\psi_{4}=\psi_{5}=\psi_{6}=\psi_{7}$ = 0). Further, we additionally use the crucial values offered by Ref. [99] in order to assess the null hypothesis of no co-integration. Furthermore, using the WALD test, we examine the short-run asymmetry based on the null hypothesis that there is no asymmetry: H0: $\eta_{1,i}=\eta_{2,i}$ = 0. In the same manner, we verify the long-run asymmetry based on the null hypothesis that there is no asymmetry: H0: $\psi_{6}=\psi_{7}$ = 0.

**Fig. 1:**
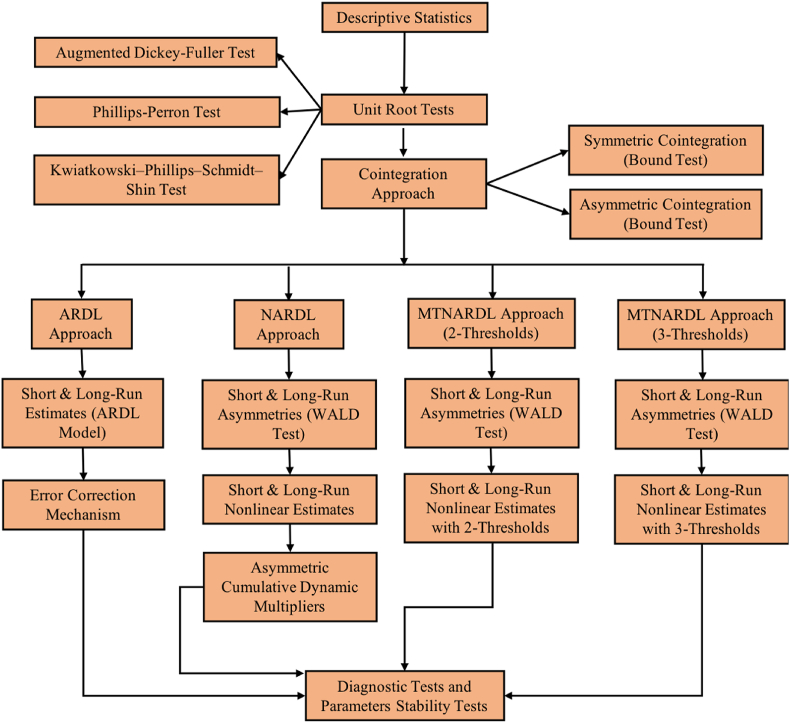
Analytical framework.

Source: Authors' Compilation

In the preceding model, the FD series was divided into two quintiles. To further evaluate the consequences of FD changes ranging from extremely positive to extremely negative, we breakdown the FD series into three thresholds at the 25th, 50th, and 75th quintiles and assess the effects of each quintile on CO2. The FD series is split into the same three thresholds as described before. Fig. 1 outline the entire analytical framework of the study.

## 4 Empirical analysis

This section comprises the model estimates outlined in the analytical framework (see Fig. 1). It begins with the model's initial estimates, including descriptive statistics, a battery of unit root tests, and cointegration. Lastly, it provides the estimates of the ARDL, NARDL, and MTNARDL approaches along with dynamic multipliers.

### 4.1 Preliminary analysis

First of all, Table 2 has a list of the descriptive statistics for each variable. The CO2 has a mean value of 6.65, with a minimum value of 5.39 and a maximum value of 7.82. In the same way, each variable's mean value is positive. Also, skewness evaluates how symmetrical the distribution of the variable is, while kurtosis shows whether the distribution is flat or has peaks. Each variable is normally distributed, given that none of them is greater than +1 or −1. This implies that there is no skewness in the distribution of the variables. However, TIN and FFC are the only factors whose kurtosis values are greater than 3 (leptokurtic). In accordance with the Jarque-Bera statistics, all of the underlying variables have a normal distribution because all of their p-values are higher than the 5 % level of significance. Other statistics may likewise be viewed in the same way.

**Table 2:** Descriptive statistics.

	lnCO2	lnFD	UB	lnTIN	HCI	lnFFC
Mean	6.65	11.44	19.36	38.38	1.65	4.57
Median	6.70	11.55	19.39	38.00	1.64	4.57
Maximum	7.82	14.19	19.97	76.00	2.17	4.58
Minimum	5.39	8.07	18.63	16.00	1.21	4.55
Std. Dev.	0.74	1.77	0.39	14.37	0.31	0.01
Skewness	−0.05	−0.29	−0.20	0.70	0.08	−1.05
Kurtosis	1.80	2.01	1.89	3.11	1.64	3.33
Jarque-Bera	2.84	2.57	2.72	3.82	3.67	3.80
Probability	0.24	0.28	0.26	0.15	0.16	0.13

Now, implementing the battery of unit root tests such as augmented Dickey-Fuller (ADF), Phillips-Perron (PP), Kwiatkowski–Phillips–Schmidt–Shin (KPSS), and Zivot-Andrew (Z-A) unit root tests by Refs. [[106], [107], [108], [109]], respectively, we subsequently evaluated the series' individual integrated properties, and the outcomes are presented in Table 3. The variables CO2, FD, and FFC are stationary at the first difference, i.e., I(1) based on the ADF and PP Tests, as shown in Table 3. TIN and Ub, on the other hand, are stationary at levels, i.e., I(0). Despite the fact that HCI is non-stationary at levels, the KPSS test revealed that HCI is stationary at the first difference, i.e., I(1). Additionally, the Z-A test is also used to identify whether variables are stationary after considering endogenous structural breaks in the data and confirming that all the variables are stationary. Since all of the variables are steady at different orders of integration, i.e., I(0) and I(1), thus unit root testing offers a compelling justification for using various ARDL estimation techniques.

**Table 3:** Battery of unit root tests.

Variables	ADF Test		PP Test		KPSS Test^@^		Z-A Test	Break Point
	Level	1st Diff	Level	1st Diff	Level	1st Diff		
lnCO2	−0.72	−7.34*	−0.72	−7.32*	0.93	0.12	−3.08**	1987
lnFD	−0.56	−6.07*	−0.56	−6.07*	0.93	0.10	3.95**	2003
lnFFC	−1.72	−8.55*	−1.55	−8.55*	0.54	0.06	−4.38**	1982
lnTIN	−3.26**	–	−3.26**	–	0.24	–	−4.27**	1989
HCI	0.16	−2.52	1.25	−2.51	0.93	0.29	−5.69*	1996
Ub	−1.07	−1.81	−10.80*	–	0.94	0.83	−4.55*	2001

Note: *&** represents the 1 % and 5 % level of significance.

After verifying the stationarity of the given variables, a bound test is used to assess the long-run connection among the variables. The outcomes are represented in Table 4 (see Panel C). The null hypothesis of no cointegration is rejected for all models because the F-statistics for the bounds test exceed the critical values of the upper bounds, establishing the long-term relationship between CO2 and its contributing factors.

**Table 4:** Results of ARDL, NARDL and MTNARDL Approaches for Fiscal Deficit-Carbon emissions nexus.

Variables	ARDL		Variables	NARDL (Single Threshold)		Variables	MTNARDL (Two Thresholds)		Variables	MTNARDL (Three Thresholds)	
	Coef.	p-value		Coef.	p-value		Coef.	p-value		Coef.	p-value
(1)			(2)			(3)			(4)		
*Panel A – Long-Run Results*											
lnFD	0.311	0.003	lnFD^+^	0.275	0.015	lnFD_25_	0.922	0.0028	lnFD_25_	2.586	0.058
lnUb	−3.224	0.000	lnFD^-^	1.594	0.000	lnFD_75_	−0.139	0.2205	lnFD_50_	1.749	0.015
lnTIN	0.010	0.000	lnUb	4.782	0.000	lnUb	2.972	0.0003	lnFD_75_	0.101	0.449
HCI	3.977 (0.039)	0.553	lnTIN	0.001	0.298	lnTIN	0.001	0.1710	lnUb	3.570	0.001
lnFFC	15.75302	0.000	HCI	−2.206 (−0.022)	0.012	HCI	−0.923 (−0.100)	0.1270	lnTIN	0.002	0.071
C			lnFFC	14.170	0.005	lnFFC	18.600	0.0100	HCI	0.185 (0.019)	0.720
			C	−148.202	0.000	C	−136.305	0.003	lnFFC	1.237	0.799
									C	−67.914	0.022
*Panel B – Short-Run Results*											
ΔlnCO_2_(-1)	−0.484	0.004	ΔlnFD^+^	−0.102	0.000	ΔlnFD_25_	−0.319	0.008	ΔlnFD_25_	−0.128	0.284
ΔlnFD	0.086	0.024	ΔlnFD^+^ (−1)	0.074	0.000	ΔlnFD_75_	0.105	0.003	ΔlnFD_25_ (−1)	−0.471	0.000
ΔlnUb	10.077	0.000	ΔlnFD^-^	−0.011	0.860	ΔlnUb	22.063	0.000	ΔlnFD_25_ (−2)	−0.291	0.018
ΔlnTIN	−0.001	0.543	ΔlnFD^−^ (−1)	0.231	0.037	ΔlnTIN	0.001	0.169	ΔlnFD_50_	0.117	0.086
ΔlnTIN (−1)	0.001	0.000	ΔlnFD^−^ (−2)	0.222	0.007	ΔHCI	2.248 (0.022)	0.000	ΔlnFD_50_ (−1)	0.500	0.000
ΔlnTIN (−2)	0.003	0.150	ΔlnFD^−^ (−3)	0.290	0.000	ΔlnFFC	2.435	0.001	ΔlnFD_50_ (−2)	0.227	0.011
ΔlnTIN (−3)	0.001	0.012	ΔlnUb	13.406	0.000	ΔlnFFC (−1)	−3.128	0.000	ΔlnFD_50_ (−3)	0.337	0.000
ΔHCI	2.00 (0.020)	0.003	ΔlnUb (−1)	8.898	0.043	ECT(-1)	−0.346	0.000	ΔlnFD_75_	0.021	0.570
ΔHCI (−1)	−1.633 (−0.016)	0.011	ΔlnTIN	0.001	0.000				ΔlnFD_75_ (−1)	−0.109	0.010
ΔlnFFC	2.068	0.016	ΔHCI	−0.090 (−0.001)	0.891				ΔlnFD_75_ (−2)	−0.050	0.127
ECT(-1)	−0.131	0.000	ΔHCI (−1)	0.586 (0.006)	0.454				ΔlnFD_75_ (−3)	−0.094	0.004
			ΔHCI (−2)	2.154 (0.021)	0.007				ΔlnUb	18.402	0.000
			ΔlnFFC	2.290	0.000				ΔlnUb (−1)	−8.838	0.005
			ΔlnFFC (−1)	−1.77	0.012				ΔlnTIN	0.001	0.000
			ECT(-1)	−0.385	0.000				ΔlnTIN (−1)	−0.001	0.008
									ΔHCI	3.309 (0.033)	0.000
									ΔlnFFC	1.342	0.035
									ΔlnFFC (−1)	1.162	0.050
									ΔlnFFC (−2)	3.972	0.000
									ECT(-1)	−0.439	0.000
*Panel C – Bound Test, Asymmetric Test, Post Estimation Tests and Stability Test*											
F-test	5.090*		F-test	5132*		F-test	5.647*		F-test	7.507*	
			W_LR_	5.409	0.000	W_LR_			W_LR_		
			W_SR_	7.347	0.000	W_SR_			W_SR_		
Adj. R^2^	0.574		Adj. R^2^	0.737		Adj. R^2^	0.543		Adj. R^2^	0.812	
***χ***^***2***^_***SC***_	4.694	0.017	***χ***^***2***^_***SC***_	2.343	0.120	***χ***^***2***^_***SC***_	0.327	0.723	***χ***^***2***^_***SC***_	2.598	0.107
***χ***^***2***^_***HET***_	0.962	0.510	***χ***^***2***^_***HET***_	2.157	0.037	***χ***^***2***^_***HET***_	0.738	0.705	***χ***^***2***^_***HET***_	1.289	0.291
***χ***^***2***^_***NOR***_	2.106	0.348	***χ***^***2***^_***NOR***_	1.209	0.546	***χ***^***2***^_***NOR***_	1.957	0.375	***χ***^***2***^_***NOR***_	0.698	0.705
***χ***^***2***^_***FF***_	0.040	0.841	***χ***^***2***^_***FF***_	0.331	0.570	***χ***^***2***^_***FF***_	0.068	0.795	***χ***^***2***^_***FF***_	0.381	0.580
CUSUM	Stable		CUSUM	Stable		CUSUM	Stable		CUSUM	Stable	
CUSUMQ	Stable		CUSUMQ	Stable		CUSUMQ	Stable		CUSUMQ	Stable	

Notes: (a) χ2SC; χ2NOR; χ2FF and χ2HET denote Breusch-Godfrey serial correlation LM Test, Jarque-Bera test for normality, Ramsey Test for functional form; Breusch-Pagan-Godfrey test for heteroscedasticity, respectively. (b) CUSUM and CUSUMQ stand for cumulative sum and cumulative sum of square. (c) Values in parenthesis are the (d) W_LR_ & W_SR_ are the WALD test for long- and short-run, respectively.

### 4.2 Linear ARDL, non-linear ARDL and MTNARDL estimates

For estimating the impact of FD on CO2 in India, we have employed a battery of advanced econometric approaches such as ARDL, NARDL with a single threshold, MTNARDL with two thresholds, and MTNARDL with three thresholds.

In Table 4 (see Column 1), the outcomes of the ARDL model revealed that the FD exerted a detrimental impact on environmental quality by stimulating CO2 in both the short and long run. However, the results of the ARDL model are unable to prove whether there is a symmetrical or some asymmetric relationship involved between FD and CO2 in the Indian context. The existing literature argues that instead of a symmetric effect, FD has an asymmetric impact on other variables [35,51,[93], [94], [95]]. Therefore, we have employed the NARDL model with one threshold and decomposed the FD into two partial sums. After the confirmation of an asymmetric cointegrated relationship among the variables, The Wald test with a null hypothesis of symmetrical association between the variables is juxtaposed with an alternative hypothesis of asymmetrical association. The W_LR_ and W_SR_ test statistics are shown in Panel C (see Column 2). The WALD test rejected the null hypothesis of symmetrical association and established the asymmetric effect of the FD on CO2 in both the short- and long-run (see Panel C and Column 2). The NARDL approach revealed that the predicted long-term coefficient of FD + has a detrimental effect on environmental quality by increasing carbon emissions. This indicates that a 1 % positive shock to FD will increase carbon emissions by 0.275 % on average. The results are consistent with those of other studies, including [57] for BRICS nations [11]; for six Asian countries: Malaysia, UAE, Turkey, Saudi Arabia, Japan, and China [48]; for Pakistan [56]; for China; and others. The feasible explanation is that the Indian government has been working to attain sustainable development over the long term while simultaneously confronting the challenges of the current turmoil, such as inflation, unemployment, etc. In addition, the recession caused significant disruptions in the production and demand sectors, as well as in economic development. To combat this circumstance, the Indian government employed an expansionary fiscal policy by increasing expenditure and reducing taxation (increase in FD) in order to increase aggregate domestic consumption spending (ADCS), promote industrial sectors, and reduce the unemployment rate. Therefore, it is evident that the increase in industrial output caused by a rise in ADCS prompted a surge in CO2. On the other hand, the predicted long-term coefficient of FD^−^ has a beneficial effect on environmental quality by decreasing carbon emissions. This indicates that a 1 % negative shock to FD will decrease carbon emissions by 1.594 % on average. These outcomes are also consistent with earlier studies, including [11,36,57]. The feasible rationale is that the Indian government implemented a contractionary fiscal policy through raising taxes and decreasing spending (decrease in FD) in order to lessen the inflation rate, which is conducive to the health of the economy. Thus, for producers, excessive taxes will result in a hike in the price of raw materials, a decline in production, ADCS, and national income, and a reduction in CO2. The aforementioned findings refute the null hypothesis (H1) and demonstrate that expansionary and contractionary fiscal policies in India have a detrimental and beneficial impact on environmental quality, respectively.

In a nutshell, the long-run coefficient values are greater when FD changes due to negative shocks than when FD changes due to positive shocks, as presented in Table 4 (see Panel A and Column 2). Alternatively, the consequence of fiscal deterioration is more pronounced, and the influence of fiscal progress is mild in terms of CO2 emission growth.

**Fig. 2:**
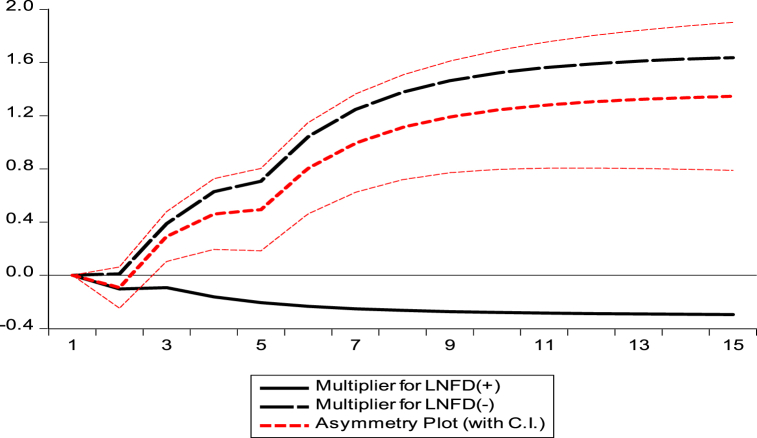
Cumulative dynamic effect of FD on CO2.

Source: Authors' calculation

Moreover, we have also constructed the dynamic multiplier graph, which supports the long-run results of the NARDL model (see Fig. 2).

The results obtained indicate that there are legitimate reasons for splitting the FD into a larger number of partial sum series in order to determine whether the magnitude of its impact differs for various levels of increase and decrease. To investigate the implications of extremely large to small variations in the FD on the CPI, we have utilized the MTNARDL model. We split the FD into two quintiles set at the 75th and 25th thresholds, i.e., Model 3 (see column 3), and into three quintiles set at the 75th, 50th, and 25th thresholds, i.e. Model 4 (see column 4), in order to investigate the effects of extreme changes in FD. In model 3, FD25 and FD75, respectively, represent extremely low and extremely high long-term changes in FD, whereas in model 4, FD25, FD50, and FD75, respectively, represent extremely low, moderate, and extreme long-term changes in FD. The results of the MTNARDL model for model 3 and 4 demonstrated that the impact of extreme large changes in FD varies from the impact of moderate and low changes, validating the long-term asymmetry. The strand of literature [35,51,[93], [94], [95]] also confirmed that FD exhibits an asymmetric impact in the long term. For robustness, the study has employed the WALD test to investigate the asymmetry between the extreme large and extreme small changes in FD in both the short- and long-run. For model 3 and 4, the WALD test rejected the null hypothesis in the long-run and documented that FD and CO2 are related to each other in an asymmetric fashion (see Panel C). This suggests that FD impacts CO2 asymmetrically in the long run compared to symmetrically in the short run. In model 3, the size of extremely low changes in FD, i.e., FD_25_ is 0.922, implying that if FD increases by 1 % in the long-run, CO2 increase by 0.922 % on average. Whereas, the long-run coefficient of extremely high changes in FD, i.e., FD_75_ is (−)0.139 but has an insignificant impact on CO2. In model 4, the size of extremely low changes in FD, i.e., FD_25_ is 2.586, implying that if FD increases by 1 % in the long-run, CO2 increase by 2.586 % on average. While, the long-run coefficient of moderate changes in FD, i.e., FD_50_ is (−)1.749. We may conclude that if FD increases by 1 % in the long-run, CO2 increase by 1.749 % on average. However, the extreme high changes in FD, i.e., FD_75_ fail to influence CO2 in the long run.

Different partial sums of the FD's coefficients have positive and substantial effects on CO2 in models 3 and 4, and these findings are reminiscent of the ARDL and NARDL models. More specifically, the size of the extremely low changes in FD is much higher than the extremely high changes in FD in both models. This suggests that as the FD rises, CO2 ascends more significantly, and when the FD lowers, CO2 declines progressively. These outcomes of the MTNARDL approach are akin to other studies such as [11,48,57], which demonstrated that FD have a long-run positive and asymmetric impact on CO2. The rationale behind these findings is quite similar to the NARDL model.

In addition, the study verified that FFC has an adverse influence on environmental quality by enhancing carbon emissions in all models. These outcomes are comparable to those of other studies [11,110,111]. These findings established that there is a significant and conclusive association between CO2 and FFC in India. The Indian economy is experiencing rapid growth, and the predominant source of energy consumption within the country is derived from fossil fuels rather than renewable sources. This reliance on non-renewable energy sources contributes to environmental degradation. The results further demonstrate that the consumption of fossil fuels plays a crucial role in influencing CO2. This association warns that the Indian government must adopt significant actions to limit the use of enormous quantities of fossil fuels through fostering the use of renewable and green sources.

TIN has an adverse effect on CO2 in all the models. This new outcome conflicts with the existing studies [74,112,113]. These two studies demonstrate how countries' TIN are focused on obtaining financial goals rather than strengthening the environment, which increases emissions. However, TIN is indispensable for the development of cutting-edge technologies that not only promote sustainable development but also reduce the consumption of resources as well as CO2 emissions. These results are in line with other studies, such as [114,115], that claim that enhancement in environmental quality is directly linked with the innovation of the energy sector. Although there is meagre literature available on the association between TIN and CO2, this outcome is corroborated by various studies that investigated the effect of TIN on levels of emissions ,[[116], [117], [118]] Furthermore, the expansion of TIN shows that technological efficacy, along with rising innovation, contributes to reducing CO2. The plausible reason is that more innovation culminates in the creation of advance technology that consumes fewer resources. The curtailment in the utilisation of resources can definitely reduce the level of CO2. Additionally, TIN is of paramount importance for the development of eco-friendly and sustainable technologies. TIN is also needed for the development of green technologies that might prevent the overexploitation of outmoded energy sources.

Likewise, there is an inverse relationship between HCI and CO2. These findings demonstrate similarities to prior research [[64], [86], [87]], indicating that HCI functions as a catalyst for promoting improvements in environmental sustainability. In essence, the presence HCI yields positive effects on various aspects, including knowledge acquisition, awareness, and efficient utilisation of natural resources, thereby enhancing environmental quality. This study presents empirical evidence regarding the impact of HC on the improvement of environmental quality. The findings suggest that this effect can be attributed to the presence of economically informed and responsible individuals who are capable of engaging in environmentally sustainable behaviours. Economic actors have the potential to enhance the environmental sustainability of their residences, communities, and countries by endorsing a lifestyle that is both sustainable and environmentally friendly.

Finally, the Ub coefficient has a substantial and negative impact on CO2 in model 1, whereas it has a positive and significant impact on CO2 in the rest of the models. According to Ref. [30], Ub has a dual impact on carbon emissions, exerting both positive and negative influences. Ub has been found to have a positive association with per capita energy consumption. This can be attributed to various factors, including the overall improvement in living standards and the subsequent rise in demand for goods and services. Additionally, the transition from traditional fuels such as straw and wood to carbon-intensive fossil fuels further contributes to this increase in energy consumption. Furthermore, the growing number of households with decreasing sizes also plays a role in this trend [119]. However, it is important to note that Ub has the potential to decrease per capita energy consumption. This can occur through the “aggregation effect” and “scale effect” that occur during intensive development, which lead to improvements in energy efficiency. For instance, Ub can result in the enhancement of service efficiency in public infrastructures, the promotion of clean energy technologies, and the facilitation of emission-concentrated treatment [120]. This outcome is similar to certain earlier studies that indicate that a rise in Ub enhances environmental sustainability [64,65,87]. As a result of experiencing high levels of Ub and the implementation of sustainable Ub rules and regulations, this high level of Ub leads to lower CO2.

Lastly, the error correction term (ECT) with CO2 as the response variable is negative and significant in all the models (see Panel C in Table 4). The negative ECT indicates that the framework is being propelled along its cointegration path in the long run. According to estimates, the coefficient of ECT, which measures the rate of adjustment, is around 63 % for model 1, 72 % for model 2, 75 % for model 3, and 57 % for model 4. This reflects the rapid correction process in model 3 compared to the moderate correction process in the remaining models.

[49] evaluated the higher accuracy of the MTNARDL model over the ARDL and NARDL models by applying the adjusted R2 value (see Table 4). Adjusting for the numerous predictors in the model, the adjusted R2 value rises only when the incorporation of extra independent variables enhances the model's predictive ability. Using Table 4, we observe that the adjusted R2 value for MTNARDL is greater than that of the ARDL and NARDL approaches. This demonstrates that the MTNARDL model is more efficient to the ARDL and NARDL models.

## 5 Conclusion, policy implications and future Agenda

Undoubtedly, throughout the past half-century, environmental quality has emerged as a significant obstacle to both economic and social endeavors. For this reason, we have examined the determinants of environmental quality in India from 1972 to 2021. More specifically, we have investigated whether the fiscal deficit is ‘curse’ or ‘haven’ for environmental quality (CO2) in India. Moreover, this study deliberated on four other predictors, comprising technological development (TIN), fossil fuel consumption (FFC), urbanization (Ub), and human capital index (HCI). In order to attain this objective, a range of econometric estimation techniques are employed to ensure the validity and reliability of the outcomes. For instance, we have employed a battery of ARDL approaches, such as standard ARDL, nonlinear ARDL, and multiple threshold NARDL approaches. In light of our research findings, we will be focusing directly on the examination of the NARDL and MTNARDL outcomes. This is due to the empirical evidence indicating the existence of asymmetric effects resulting from FD on CO2 emissions in India.

The following is an outline of the study's outcomes. First, according to the ADF, PP, and KPSS unit root tests, each of the variables is stationary at diverse order of integration, i.e., I(0) and I(1). As a result, unit root testing significantly contributes to the justification for using distinct ARDL estimation techniques. In addition, the Z-A test is also used to identify whether variables are stationary after considering endogenous structural breaks in the data and confirming that all the variables are stationary. Second, the long-term relationship between CO2 and its drivers is confirmed by F-statistics. Third, the outcomes of the ARDL model revealed that the coefficient of the FD exerted a positive impact on CO2 in both the short- and long-run. Fourth, the NARDL approach reveals that the consequence of fiscal deterioration is more pronounced, and the influence of fiscal progress is mild in terms of CO2 emission growth. Fifth, the outcomes of the MTNARDL approach revealed that the size of the extremely low changes in FD is much higher than the extremely high changes in FD in both models. This suggests that as the FD rises, CO2 ascends more significantly, and when the FD lowers, CO2 declines progressively. In a nutshell, FD has a long-run positive and asymmetric impact on CO2 in India, thus, we may conclude that FD is considered the ‘curse’ for CO2 in India. Sixth, TIN, HCI, and Ub has a detrimental effect on CO2, whereas the FFC has stimulated CO2 in India. Lastly, The MTNARDL model's supremacy over the ARDL and NARDL models is shown by its high adjusted R2 value.

## Policy implications

This research study offers significant policy implications for a range of stakeholders, including environmentalists, macroeconomic policymakers, and organisations dedicated to environmental sustainability, in order to advance the cause of a sustainable and ecologically sound environment. The findings indicate that there is a negative relationship between fiscal deficit and environmental quality, highlighting the importance of implementing a green fiscal policy. It is imperative for the government to develop and implement effective mechanisms for green fiscal policy. It is recommended that the government implement carbon taxes, green subsidies, and green public investment measures. The implementation of a carbon tax policy has the potential to effectively mitigate greenhouse gas emissions by establishing a monetary value on carbon emissions. The underlying concept of carbon taxation is to establish a financial stimulus for individuals, businesses, and governments to diminish their carbon emissions by incorporating the societal expenses associated with their actions. Further, the outcomes of the study reveal that the response of CO2 is positive due to change in TIN. This implies that TIN in environmental conservation/sustainability can lead to healthier living conditions, adoption of renewable energy resources, creation of new job opportunities, sustainable urbanization, and cultural preservation. Furthermore, the findings indicate that there is a negative interaction between HCI and CO2, implying that HCI has a positive impact on environmental quality. The prioritisation of human capital development by the government is crucial in enhancing environmental quality through the enhancement of knowledge, awareness, and efficient resource utilisation, among other factors. The study may be assessed by policymakers in order to formulate policies pertaining to Sustainable Development Goal 13.

## Future research

The current study has investigated the efficacy of fiscal policy in promoting environmental quality in India. However, it is recommended that future research should explore the implementation of green fiscal policy instruments, including carbon taxes, green subsidies, and green investments, in order to assess their effects on environmental quality. Further, the current investigation has taken into account carbon emissions as a surrogate measure for environmental quality. In addition to carbon emissions, future research endeavours may incorporate the examination of ecological footprints and other greenhouse gases, including methane, nitrous oxide, and fluorinated gases, in order to develop policy implications that are both practical and suitable. In addition, incorporation of institutional quality and political instability variables as determinants of environmental quality could be considered in future research endeavours.

## Author contribution statement

Mohammad Asif: Conceived and designed the experiments; Analyzed and interpreted the data; Wrote the paper. Vishal Sharma: Analyzed and interpreted the data; analysis tools or data. Hari Prapan Sharma; Contributed reagents, materials, analysis tools or data. Hamad Aldawsari; interpreted the data, Wrote the paper. Showkat Khalil Wani: materials, analysis tools or data; Wrote the paper. Sunil Khosla; Conceived and designed the experiments, Wrote the paper. Vinay Joshi Chandniwala, Contributed reagents, Wrote the paper.

## Data availability statement

Data will be made available on request.

## Declaration of competing interest

The authors declare that they have no known competing financial interests or personal relationships that could have appeared to influence the work reported in this paper.

## Footnotes

1 For detail understanding as well as derivation of ARDL approach, please refer to [99].

2 For detail understanding as well as derivation of ARDL approach, please refer to Ref. [100].
